# Postrenal Failure due to Urinary Stones Associated with Acute Viral Gastroenteritis: Three Case Reports

**DOI:** 10.1155/2016/1375923

**Published:** 2016-10-26

**Authors:** Satoru Kira, Takahiko Mitsui, Hidenori Zakoji, Tadashi Aoki, Norifumi Sawada, Tatsuya Miyamoto, Masayuki Takeda

**Affiliations:** Department of Urology, University of Yamanashi Graduate School of Medical Science, 1110 Shimokato Chuo, Yamanashi, Japan

## Abstract

Acute gastroenteritis with viral infection in infants causes severe diarrhea and often results in acute renal failure due to severe dehydration. However, a viral infection, particularly rotavirus, rarely induces postrenal failure due to bilateral stones in infants. Herein, we report three cases of postrenal failure in infants due to bilateral ureteral stones induced by acute gastroenteritis with rotavirus. Following immediately nephrostomy, chemical dissolution therapy succeeded to treat postrenal failure. Immediate nephrostomy for the release of upper urinary tract obstruction combined with urinary alkalization as a chemical dissolution therapy should be considered in such cases.

## 1. Introduction

Acute gastroenteritis with viral infection is the most common cause of severe diarrhea in infants and young children, often eliciting severe dehydration and prerenal failure [1, 2]. Recently, it has been reported that acute gastroenteritis with viral infection induced acute postrenal failure due to bilateral obstructive ureteral stones in infants [3–5]. However, the urological management strategy for postrenal failure due to acute gastroenteritis associated with viral infection has not yet been adequately discussed. Herein, we report three cases of postrenal failure in infants due to bilateral obstructive ureteral stones associated with acute gastroenteritis and discuss urological management for such cases.

## 2. Case Presentation

A summary of the clinical and laboratory data for all three cases is shown in Table 1.

*Case 1*. A 28-month-old boy with a 4-day history of severe diarrhea and vomiting was referred to our hospital due to acute renal failure. Ultrasound sonography (USG) and computed tomography (CT) revealed bilateral hydronephrosis and ureteral stones. Thus, he was diagnosed with postrenal failure due to bilateral ureteral stones. In order to treat the postrenal failure, a percutaneous left nephrostomy tube was inserted under general anesthesia. On the 4th day after starting hydration and urinary alkalization, sandy stones were drained through the nephrostomy tube. The level of creatinine and blood urea nitrogen returned to normal range in postoperative day (POD) 3. At one week after nephrostomy tube insertion, a CT scan showed vanishing bilateral hydronephrosis and stones and the nephrostomy tube was extracted. He was discharged on POD 12.

*Case 2*. A 13-month-old boy with a 5-day history of severe diarrhea and vomiting caused by rotavirus and gastroenteritis was referred to our hospital due to severe dehydration and acute renal failure. USG and CT showed bilateral mild hydronephrosis and ureteral stones. Under general anesthesia, a percutaneous right nephrostomy tube was inserted for the drainage of urine. Sandy stones were drained through the nephrostomy tube on the 3rd day after initiating hydration and urinary alkalization. At 2 weeks after nephrostomy tube insertion, a CT scan showed no evidence of stones and the nephrostomy tube was extracted.

**Figure 1:**
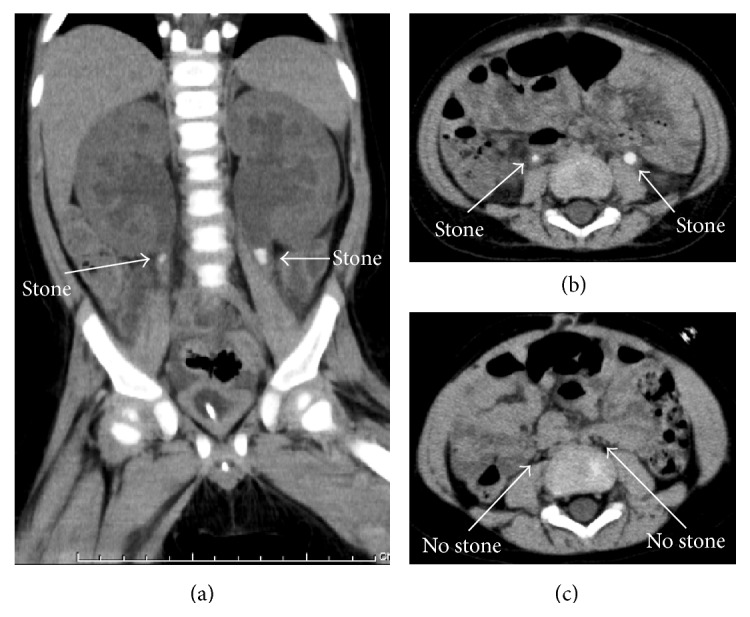
Computed tomography (CT) images for Case 3 are shown. (a) A coronal CT section revealed ureteral stones with hydronephrosis in the bilateral ureters (arrows). (b) A transverse CT section revealed ureteral stones with hydronephrosis in the bilateral ureters (arrows). (c) A transverse CT section after treatment is shown. The ureteral stones vanished and the hydronephrosis was resolved (arrows).

*Case 3*. A 15-month-old boy with a 5-day history of severe diarrhea and vomiting was referred to our hospital due to anuria and acute renal failure. USG and CT showed bilateral mild hydronephrosis and ureteral stones (Figures 1(a) and 1(b)), indicating postrenal failure caused by the obstruction of bilateral ureters with urinary stones. As in the other two cases, a percutaneous right nephrostomy tube was inserted under general anesthesia. Following treatment with hydration and urinary alkalization, sandy stones were drained out through nephrostomy tube on the 1st day. At 10 days after nephrostomy tube insertion, a right percutaneous antegrade pyelography showed that the obstruction due to ureteral stones was vanishing and the nephrostomy tube was extracted. At 3 months, a CT scan showed no evidence of stones (Figure 1(c)).

## 3. Discussion

Postrenal failure due to bilateral obstructive ureteral stones after acute gastroenteritis with viral infection in infants has been mainly reported in Japan [3–6]. In most cases, ammonium acid urate (AAU) was detected in the analysis of stone component [7]. Considering our cases, sandy stones were confirmed to consist of AAU in two patients and were assumed to consist of AAU in another patient given the clinical course. In fact, following nephrostomy as treatment of upper urinary tract (UUT) obstruction and urinary alkalization of urine, UUT obstruction caused by stones was resolved within a few weeks in our cases.

The formation of AAU stones is induced by the combination of aciduria, hyperuricemia, and hyperammonuria [8]. The mechanism underlying the formation of AAU stones in patients with laxative abuse has been described as follows. Severe gastrointestinal loss of water and electrolytes induces acute volume depletion and intracellular acidosis and increases excretion of urinary ammonium. The low urinary sodium owing to severe diarrhea allows for the coupling of urate with the abundant ammonium ion. This coupling, combined with at least a transient state of ammonium urate supersaturation, promotes the formation of AAU stones [9]. This mechanism is similar to that for the formation of stones induced by rotavirus in infants. However, the precise pathogenesis remains unknown. Furthermore, no reports exist regarding the urological management of postrenal failure due to obstructive ureteral stones associated with viral infection and gastroenteritis. Thus, we believe that the following discussion of urological management strategies is necessary for this clinical issue.

First, the presence or absence of hydronephrosis should be assessed by using USG during the initial assessment for renal failure associated with acute viral gastroenteritis in infants. If bilateral hydronephrosis is detected, postrenal failure due to bilateral obstructive ureteral stones should be considered. If not, it is better to consider prerenal failure due to dehydration. In most cases, bilateral hydronephrosis is detected on USG or CT. Considering radiation exposure, quickness, and cost, USG should be recommended first for the detection of UUT obstruction.

Second, if bilateral hydronephrosis is detected, percutaneous nephrostomy should be considered. In these cases, unilateral nephrostomy is enough to recover renal function from acute post renal failure. As unilateral renal function is being recovered, general conditions including volume depletion and metabolic acidosis are normalizing promptly. Considering the mechanism of AAU stone formations in these cases, recovery from metabolic acidosis leads resolution of bilateral AAU stones by normalization of urinary PH. Treatments for releasing UUT obstructions from 32 cases of postrenal failure due to ureteral stones associated with acute viral gastroenteritis are reviewed in Table 2. In about half of these cases, nephrostomy was indwelled. For this reason, the nephrostomy tube may result in a definite and quick release of the UUT obstruction and enable antegrade pyelography afterwards. Among the other treatment options, a ureteral stent may be considered as the most noninvasive approach. However, a nephrostomy tube was better than a ureteral stent in our cases, because patients were infants with an unstable condition and immediate intervention was required. For the insertion of ureteral stents in infants, a special size should be prepared. In cases of peritoneal dialysis or hemodialysis, the diagnosis of postrenal failure may be delayed before treatment. Given these considerations, nephrostomy should be immediately considered in infants due to a definite and quick release of the UUT obstruction and the use of a catheter for later antegrade pyelography.

Third, the urine should be alkaline. In most cases with ureteral stones caused by viral gastroenteritis, analysis of component of stones showed AAU [7]. Given the specificity of AAU, urinary alkalization is more effective as a chemical dissolution therapy [8]. In our review, urinary alkalization was performed in about one third cases (10/32). In all these cases, AAU stones were confirmed as analysis of stones and resolved after alkalization of urine. Furthermore, considering the mechanism of AAU stone in infants with rotavirus, the treatment of metabolic acidosis may prevent the formation of an AAU stone [10]. However, in most cases, urgent intervention is required because of life-threatening postrenal failure in infants. Therefore, urinary alkalization as a chemical dissolution therapy should be considered after resolving the UUT obstruction.

In conclusion, acute gastroenteritis with viral infection, particularly rotavirus, rarely induces postrenal failure due to bilateral stones in infants. The mechanism of stone formation remains unknown. In such cases, nephrostomy should immediately be considered as a treatment option for a definite and quick release of the UUT obstruction, followed by a chemical dissolution therapy.

## Figures and Tables

**Table 1:** Summary of the clinical and laboratory data for the three cases.

Clinical and laboratory data	Case 1	Case 2	Case 3
Age (months old)	28	13	15
Interval from outbreak	4 days	5 days	5 days
Rotavirus antigen in EIA	Positive	Not measured	Positive
Laboratory data in serum			
BUN (mg/dL)	107.9	84.3	40.5
Cre (mg/dL)	6.38	5.25	2.34
UA (mg/dL)	15.1	17.3	10.0
Na (mg/dL)	132	127	135
Analysis of stones	Not investigated	AAU	AAU

EIA, enzyme immunoassay; BUN, blood urea nitrogen; Cre, creatinine; UA, uric acid; Na, sodium; AAU, ammonium acid urate.

**Table 2:** The treatments for releasing UUT obstruction in 32 cases of postrenal failure due to ureteral stones associated with acute viral gastroenteritis.

Treatment for releasing obstruction of UUT	Numbers
Percutaneous nephrostomy (including bilateral)	16
Exclusion of stones	7
Ureteral stent (including bilateral)	4
Peritoneal dialysis	3
Hemodialysis	5

## Abbreviations

EIA: Enzyme immunoassay
BUN: Blood urea nitrogen
Cre: Creatinine
UA: Uric acid
Na: Sodium
AAU: Ammonium acid urate
USG: Ultrasound sonography
CT: Computed tomography
POD: Postoperative day
UUT: Upper urinary tract.
